# Access to healthcare for undocumented migrants in France: a critical examination of State Medical Assistance

**DOI:** 10.1186/s40985-016-0017-4

**Published:** 2016-08-03

**Authors:** Jean-Marie André, Fabienne Azzedine

**Affiliations:** 1 EHESP French School of Public Health, Rennes, Sorbonne Paris Cité, Rennes, France; 2 CNRS (National Center of Scientific Research), UMR CRAPE (Research Center on Politic Action in Europe), Rennes, 6051 France

**Keywords:** Health access, Undocumented migrants, Health protection

## Abstract

In France in 2012, of the total population of 65.2 million, 8.7 % were migrants. After being the third principal host country, France is now the 6th highest host country in the OECD. Since the 1980's numerous Acts have been passed by parliament on immigration issues.

In 2000 the Universal Health Cover (Couverture Maladie Universelle) was created as health coverage for all residents of France. At the same time the State Medical Assistance (Aide Médicale de l’Etat) was created as health protection for undocumented migrants. Since the creation of this scheme, it has been the object of many political debates which call it into question, on account of its cost, perceived fraud, and the legitimacy of a social protection for undocumented migrants. Recently, access to State Medical Assistance has been made difficult by introducing conditions of residence and financial contributions.

After a reports’ analysis on institutional, associative, research studies and European recommendations, we note that all reports converge on the necessity of health protection for undocumented migrants. The major reasons are humanitarian, respect of European and International conventions, for public health, and financial. Moreover, fraud allegations have proved to be unfounded. Finally, State Medical Assistance is underused: in 2014 data from Médecins du Monde shows that only 10.2 % of undocumented migrant patients in their health facilities have access to this scheme.

We conclude that the political debate concerning the State Medical Assistance should be about its under-utilisation, its improvement, its merger with the Universal Health Cover, and not its elimination. Moreover, the current debates regarding this scheme stigmatize this population, which is already precarious, making it more difficult for migrants to access healthcare, and generally, weaken national social cohesion.

## Background

The French health system is generally considered to be of a high quality in comparison with other national systems [1]. However, the results present a number of inadequacies, in particular regarding unequal access to care. This is the case both at the point of entry to the system, and during later healthcare procedures in the varying quality and pertinence of the medical acts carried out [2]. In terms of access to primary care, the people who experience the greatest difficulties are those who are the most economically insecure. This is because they are less capable of paying the amounts outstanding after social security cover. The result is often a failure to take up treatment, which concerns more than one in three in some categories of the population [3].

Migrants^1^ represent one of these categories. Over 20 % of migrants do not possess optional health insurance,^2^ compared to about 5 % of the general population. A distinction is made between migrants who are in a regular situation (documented) in the country and those who are not (undocumented migrants). The former can claim common law benefits, whereas the latter are only eligible for a specific benefit called State Medical Assistance - Aide Médicale de l’Etat.

This benefit, which dates from 2000, is the object of a great deal of criticism. Following an overview and a close look at the situation of migrants in terms of health in France, we present the State Medical Assistance, examine why it is the focus of such criticism, and set out the main questions it raises. This development leads us to question the interest of this benefit and then present the methodology formulated to try and provide a response. The lessons learned are identified and discussed. Lastly, we make several proposals for moving towards a more satisfactory solution.

### Immigration in France and health data on migrants

Migrants’ health as a research subject has not been well developed in France. Several explanations have been put forward, i.e. the lack of health statistics including nationality criteria; the irrelevance of making a distinction between French nationals and non-nationals in the “republican” model; and concerns about encouraging a xenophobic discourse, or of being categorized as “communitarian”. These different arguments have had the effect of challenging the legitimacy of such a study subject in the social science field [4].

However, France has an established, rich and significant history of migration. At the start of the 20^th^ century, 1.1 million migrants lived in mainland France, which is about 3 % of the population. In 1954, numbers had grown to 2.3 million, and 4.2 million by 1990, representing respectively 5.2 and 7.5 % of the population. After stabilizing during the 1990s, the immigrant population grew again after 1999, reaching 5.7 million for a total of 65.2 million inhabitants, which is 8.7 % in 2012 [5].

Until the mid-1970s, immigration flows were mostly male, satisfying labour requirements to rebuild the country after the Second World War and the three decades of economic growth that followed. Since 1975, the number of women migrants has continued to grow. In 2012, 51 % of migrants were women, compared to 44 % in 1968. In addition, migrants’ geographical origins are becoming more diverse. In 2012, 43 % of migrants, or 2.5 million people, were born in Africa. Those born in the Maghreb represented 30 % of the total. This proportion has remained steady since the 1980s. Immigration from sub-Saharan Africa is more recent and mostly concerns countries previously under French administration.

Despite relatively stable entry flows, policy on immigration and living together is regularly subject to doubts and crises. The main reason for immigration was for employment purposes, but since 1974 family reunification has been the predominant motive. In 2014, of 219,374 first residence permits delivered, 45 % were connected to a family motive and only 9 % to work [6]. Starting from the 1980s, immigration has been the object of numerous laws reflecting the views of alternating governments [7]. To take recent examples, 2015 saw a draft law on reforming asylum, a draft reform of the code for foreign nationals’ entrance and residency, and a draft constitutional act to protect the nation concerning the procedure for revoking the citizenship of bi-nationals. One of the reasons behind this increased legislation is the growth since the 1980s of the extreme right-wing party, which is now one of the three main players in the French political arena. This new configuration has contributed to dramatizing the topic of immigration within public debate, presenting it is a problem or even a threat [8].

France is no longer one of the main three destination countries, ranking only sixth [9]. Despite its long tradition as a host country, the OECD (Organization for Economic Co-operation and Development) indicates that France faces persistent difficulties. The most obvious domains are those in which French indicators are unfavourable compared to countries in Europe or the OECD area, i.e. migrants’ access to the labour market; the relative poverty rate^3^; and the education outcomes of their children [10].

These data on living conditions show how important it is to develop research on the determinants of migrants’ health in France. Studies currently available show that whereas migrants’ health status was generally better than that of non-migrants during the 1990s and 2000s, it appears to have declined since. This evolution is also confirmed in the other major immigration countries. The “healthy migrant effect” (their better health status, mostly due to the selective factors of immigration) disappears with the duration of residence in the host country [11]. More precisely, health status now appears to be worse for first-generation and female migrants. Significant differences are also apparent depending on the country of origin. In terms of health care, this takes the form of fewer requests for preventative and non-hospital care. Added to these health factors, the financial problems that migrants frequently face, because they are less integrated into the socio-economic fabric and possess more fragile social connections, clearly exacerbate these difficulties [12].

In recent years, research has been undertaken to understand the importance of social capital and socio-economic conditions [13], the diversity of situations depending on geographic origin [14], and the impact of discriminating professional representations [15] on migrants’ health.

However, few studies have centred on the health of undocumented migrants. The data available mostly relate to the health determinants connected to living conditions. Most of the information on these subjects comes from reports by associations. Thus, according to the latest report by Médecins du Monde, 53 % of their patients were in an irregular situation. Only 6 % occupied personal accommodation, 98 % had income below the poverty threshold^4^ and one third had no income whatsoever [16].

### State Medical Assistance and the main points for discussion

In France, State Medical Assistance is part of social welfare policy, which represented 32.1 % of GDP in 2013. The country’s policy places France second in the Europe Union for this indicator, behind Denmark (33.1 %), and fairly far ahead of the European average, which is around 28.5 % [17]. Taken as a whole, the share devoted to health represents 34.6 %.^5^

Considering total consumption of care and medical goods (which came to 191 billion euro in 2014) public intervention covers 78 % of expenditure. The remaining 22 % is covered by optional health insurance (13.5 %) and by patients themselves (8.5 %) [18]. The management of the public cover is not done by the state, but like others European systems, based on the Bismarck model, by the social security bodies. Unlike the UK and Nordic countries, it is based on a set of social security funds managed by social partners.^6^

The largest share (98.2 %) of health care expenditure that is reimbursed by the social security is provided regardless of patients’ income. Some means-tested national solidarity allowances are available to improve access to care for the poorest (1.8 %). These allowances are: complementary universal health cover – couverture maladie universelle complémentaire (CMUc),^7^ which covers health expenditures that are not fully met by the obligatory health insurance; health cover assistance, which provides financial assistance for purchasing complementary health insurance for people whose income is just above the threshold for receiving CMUc; and State Medical Assistance, the focus of this article.

State Medical Assistance is a social benefit created at the same time as universal health cover – couverture maladie universelle (CMU) – and which came into force in early 2000 to help combat exclusion. It concerns foreign nationals who live in irregular situations in France and are subject to financial difficulties. To receive this assistance, beneficiaries need to have been on the national territory for over three months and have an annual income of less than 8,645 euro. For a couple, the threshold is 12,967 euro. Several supporting documents are required to prove the identity of the applicant, an address of domiciliation and over three months’ presence in France. Dependent people can also benefit from State Medical Assistance (i.e. partners and children). Children under 18 can claim the allowance if their parents are in France in an irregular situation and not eligible for the allowance.

Another measure existed prior to State Medical Assistance, a local medical assistance. This benefit aimed to provide social protection for all people living in poverty and insecure conditions. Note that until 1993, regular residency was not required to benefit from this local medical assistance; the only condition was to have lived in France for three years [19]. The Act of 24^th^ August 1993 on immigration control created a distinction between, on the one hand, foreign nationals in regular situation, who could continue to claim medical assistance in the same way as nationals, and on the other hand, foreign nationals in irregular situation, who could only receive such assistance provided they had been present on the territory for at least three years.^8^ The creation of CMU in 2000 gave French people and foreign nationals living legally in France but not affiliated to a particular health insurance scheme based on socio-professional criteria, the right to claim health insurance for basic welfare and, depending on income, for complementary insurance. At the same time, the new measure separated undocumented migrants into a specific category by giving them the right to social cover in the form of the State Medical Assistance.

Currently, State Medical Assistance covers all medical treatment resulting from illness or childbirth within tariffs determined by the Social Security.^9^ Fees are not paid out in advance. All healthcare professionals are obliged to accept State Medical Assistance beneficiaries. The period of entitlement is one renewable year.

For undocumented migrants who cannot justify minimum residency to benefit from State Medical Assistance, an overriding measure exists to cover emergency care, i.e. care “the absence of which would be life-threatening or could lead to a serious, long-term alteration of the health status of the person or unborn child” [20]. Treatment to avoid the propagation of disease (e.g. tuberculosis) is also concerned.^10^ In total, emergency care only represents 12.5 % of the amount spent on State Medical Assistance [21].

The non-governmental organization (NGO) Platform for International Cooperation on Undocumented Migrants (PICUM) attempted in its 2007 report [22] to rank European Union member states in terms of their medical cover for undocumented migrants. The report categorizes five main situations: countries where medical care is provided for payment (Austria, Sweden), those that offer free medical care in very limited cases (Hungary, Germany), those offering broader cover but with more restrictive legislation that leads to ambiguity (United Kingdom, Portugal), those who have set up a parallel administration system (France, Belgium), and those where access to care is open to everyone (Italy, Spain). Note that the reforms in Spain in 2012 changed the categorization of this country [23].

In France, State Medical Assistance is hotly debated and regularly subject to criticisms calling for stricter eligibility criteria or, more radically, its revocation. These criticisms are based on four main arguments. The first concerns the very legitimacy of assistance, paid for by the national community, to people who do not have the required conditions for staying on French territory and who are sometimes suspected of having left their country of origin for invalid reasons to come to France.^11^ The second argument is that the French measure appears to be particularly generous and that it finances a fairly broad range of goods and services that significantly exceeds emergency requirements. International comparisons are made to illustrate this situation [24]. The generally more restricted scope for receiving public assistance in other European countries is highlighted [25]. The third reason, partly connected to the second, is uncontrolled expenditure. Recent reports indicate that the cost of State Medical Assistance rose 26 % from 2010 to 2014, while total medical consumption only increased 9.8 % during the same period [25]. The fourth and final argument concerns fraudulent claims that are granted unwarranted benefits [25]. We will look more closely at these different points later in this article.

Depending on the political leanings of recent governments, these criticisms have sometimes resulted in more rigorous conditions for allocating State Medical Assistance. Thus, from 2003, the condition of three months’ uninterrupted residency was established, and the decrees of 2005 removed the possibility of providing sworn statements, except for financial means. However, one of the proposals most frequently mentioned is beneficiaries’ financial participation. This highlights the principle of “accountability” that should apply to all welfare beneficiaries. It is also based on some opinion polls indicating that over 60 % of French people think that those without right to residency should not escape this principle.^12^ Thus, in 2011, an “access fee” of 30 euro was established. This measure was revoked the following year and the free-of-charge principle was re-established, in line with commitments made by the new French president. It is possible that this “access fee” may be reactivated in years to come, and private bills have already moved in this direction [25]. Similarly, regular calls are made for a “health basket” restricted to emergency care, life-saving care and treatment to avoid epidemics. Another demand is more severe sanctions for those recognized as guilty of fraud and for healthcare professionals who encourage them [26].

### Questioning and methodology

As mentioned above, State Medical Assistance, as an assistance benefit aimed at undocumented people, is subject to hot political debate. However, beyond the controversies, it is worth questioning the concrete impacts of this public intervention. To do so, we examine several questions, considering the current situation as well as possible changes. The questions are as follows: How much does State Medical Assistance really cost? Who are the beneficiaries? Is this allowance correctly implemented? What are the impacts in terms of public health? The answers to these questions give us the material to discuss the legitimacy of the measure and suggest ways of improving it.

Our research is mostly based on an analysis of reports and contributions of different actors involved in the topic of migration. We examined the work of six major analysis producers: French parliament, French general state inspectorates, human rights institutions, research bodies,^13^ humanitarian associations, and the World Health Organization.

Two reports on State Medical Assistance written by deputies were published in 2011 and 2015. They make a technical and political diagnosis of its evolution.

At the request of ministries, the General Inspectorate of Social Affairs (IGAS) and the General Inspectorate of Finances (IGF) have published three reports over the last ten years with a particular focus on the cost of the measure.

The Rights Defender and the Consultative Council on Human Rights have produced three analyses of the questions raised by State Medical Assistance regarding respect for human rights.

Among the humanitarian associations, “Médecins du Monde”, which manages health centres in around twenty major towns at which over 90 % of the patients are migrants, produces two annual reports as part of its observatory on access to rights and healthcare. These reports provide additional information on people’s health status. Documents on the subject are also produced by other associations and we looked at the work done by “Groupe d’Information et de Soutien aux Immigrés” (GISTI) – Group of information and support for migrants and the “Comité Médical des Exilés” (COMEDE) “Medical committee for exiled persons”.

Academic research on the health issues facing undocumented migrants is relatively scarce. However, we explored francophone scientific literature by entering the terms “health care” and “migrants” and synonyms into the databases CAIRN and TERRA (“Travaux Etudes et Recherches sur les Réfugiés et l’Asile” – Studies and researches on refugees and asylum). We also examined scientific literature in English, with searches in Pubmed and Academic Search Premier.

Our analysis is qualitative in nature, integrating different sub-themes included in our research questions, based on the political debate: the cost of the State Medical Assistance, the presentation of the beneficiaries, the implementation of this scheme and its impact on public health.

## Main results

### How much does State Medical Assistance cost?

In 2014, expenditure relating to State Medical Assistance totalled 831 million euro for the all undocumented migrants [21]. This represented 0.49 % of expenditure on social welfare devoted to covering the consumption of medical goods and services [18]. The average annual expenditure per beneficiary came to 2,823 euro.^14^ This average figure hides significant disparities, since statistic analyses show that 75 % of beneficiaries incur expenses below 1,000 euro and 3 % above 10,000 euro. Although average expenditure per person has been relatively stable over the last few years (it was 2,846 euro in 2007), we observe a clear increase in overall expenditure, from 661 million euro in 2010 to 831 million in 2014, or an increase of 26 %. As we shall see, this increase is mostly due to a rise in the number of beneficiaries. Note also that, since its creation, the budget devoted to State Medical Assistance has been consistently underestimated compared to actual needs [27].

In terms of the organization of expenditure, hospitalization costs represent 70 % of the overall amount, compared to less than 50 % for those insured under the social security system. This observation can be related to the progression of emergency care, generally requiring a hospital stay, which went up 38 % from 2010 to 2014.

### Who are the beneficiaries?

At the end of 2014, State Medical Assistance counted 294,000 beneficiaries, up from 230,000 in 2010, an increase of 28 % [25]. The annual renewal rate of beneficiaries is 10 %. This rate is in line with the principle that State Medical Assistance is not designed to assist people for long periods. The beneficiaries are not evenly distributed around France: 40 % are concentrated in two health insurance funds in the Paris region: Ville de Paris and Seine-Saint-Denis (northern suburbs). Two thirds of beneficiaries are registered with 8 health insurance funds in the Paris region. The other regions with the highest numbers are Marseille and Lyon.

The vast majority of beneficiaries are single people. In Paris, only 10 % of female beneficiaries are entitled through their partner. Claimants are also relatively young, with two-thirds aged from 20 to 45 years old. Men are significantly more numerous than women: 57 and 43 % respectively.

Few recent data are available on the geographical origin of beneficiaries. A study undertaken in 2008 indicates that 43 % come from sub-Saharan Africa, 20 % from the Maghreb, and 17 % from Asia [28]. More recent partial analyses indicate that the first group clearly represent a larger proportion today, with an increase in beneficiaries from Ivory Coast and Mali [25].

### Is State Medical Assistance correctly implemented?

This question involves looking at whether all people in a situation to rightfully claim State Medical Assistance can effectively access the assistance and, on the contrary, whether unwarranted benefits are paid out following fraudulent applications. Concerning the first point, it is widely recognized that not all people eligible for State Medical Assistance carry out the necessary steps for claiming it [27]. As a result, the number of beneficiaries recorded at the end of 2014 (294,000) is significantly below the estimated number of undocumented migrants, estimated at between 300,000 to 400,000 depending on the source^15^ [29], bearing in mind that it is particularly difficult to establish statistics in this domain [30]. According to analyses of Médecins du Monde patients, non-take-up can be explained by four major factors [16]: lack of knowledge of the measure (37 %); administrative requirements (33 %) that call for documents that are sometimes difficult to obtain (27 % of the people studied had no fixed address); language difficulties (20 %), which limit efficient communication with the administration; and fear of being called in for questioning (30.5 %), which restricts people’s movements.

Another factor is that accessing the benefit depends on the practices and professional representations of the agents handling applications. Depending on the case, these agents may make more or less restrictive appraisals of applicants’ admissibility [31]. These practices and representations can sometimes involve a climate of suspicion concerning foreign nationals [32]. This kind of difficulty is illustrated by the activity of the Médecins du Monde healthcare centres, which show that only 10.2 % of patients eligible for State Medical Assistance have effective access [16]. In some cases, eligibility does not necessarily guarantee the possibility of receiving care because in certain situations treatment may be refused [33], mainly by doctors in the private sector.^16^ Healthcare professionals in this sector sometimes esteem that treating State Medical Assistance patients introduces too many complications into their activity [27].

Fraud is particularly difficult to evaluate by definition. Studies on the subject point to three main areas of fraud: declaration of identity, declaration of residence, and declaration of income [34]. Recent analyses show that they probably respectively represent 6, 25 and 63 % of prejudice [35]. To combat these frauds, 160 agents are specifically responsible for carrying out checks a priori, when the application is made, and a posteriori [35]. According to the “Caisse Nationale d’Assurance Maladie” – National Fund Health Insurance, 54 fraudulent applications were detected in 2014, amounting to a total prejudice of 130,000 euro, which is negligible compared to the overall expenditure [25]. These results are in line with what was observed several years earlier in the Seine-Saint-Denis area, which showed that financial prejudice only represented 0.12 % of expenditure [34].

Ultimately, it therefore appears that, although it is important to remain vigilant regarding the proper use of the benefit in order to guarantee its legitimacy, fraud does not constitute a convincing explanation for the increase in expenditure. To determine whether this measure is efficiently implemented should rather involve examining its under-utilization and its real impacts on people’s health.

### What are the impacts on public health?

The primary benefit of State Medical Assistance in terms of public health is to open up access to health for almost 300,000 people, even though this access is not effective for all those who may need it.

Data on the health status of State Medical Assistance beneficiaries are limited, but some information exists on the motives for accessing different services. This accessibility, made possible thanks to State Medical Assistance, informs us about the needs it responds to. The first point of interest is the number of hospital admissions. As pointed out above, this proportion is higher than for the general population. This implies that State Medical Assistance covers the cost of care for people with serious health issues, which, in its absence, could threaten their lives. However, it also informs us that the implementation of this measure does not guarantee optimal access to primary health care. This can lead to more complicated or worse conditions.

A study of the diseases endured by hospitalized State Medical Assistance beneficiaries provides more precise information on this aspect [27]. This section of the population suffers from similar diseases to people covered by the national insurance scheme, but with a higher prevalence of HIV, hepatitis C and tuberculosis. Hospital stays for pregnancy, childbirth and female genital infections are two to four times greater than for people covered by national insurance. Along with the inadequacies of primary care cover, associations point to the traumatic stress suffered by patients following violent events in their countries of origin.

Another indication of the possible impacts of State Medical Assistance can be obtained by assessing the health conditions suffered by those who do not claim it. Few data are available on this subject, but we can draw from the experience of the Médecins du Monde health centres [16]. This shows that over 90 % of undocumented patients who came to the health centres in 2014 had no welfare cover. Analysis showed that 62 % suffered from a chronic disease requiring regular check-ups, 42 % had delayed seeking care and 26 % required urgent treatment. These alarming figures should be compared with the under-utilization mentioned above. They indicate that any measure that tends to improve conditions for access to care can only have positive impacts on consolidating these people’s health and limiting a worsening of their situations.

Lastly, State Medical Assistance contributes to public health by restricting the spread of contagious diseases. Although migrants arrive in France in relatively good health, the very insecure situations they live in often expose them to diseases that require rapid, effective treatment to avoid propagation.

To sum up, and based on complementary qualitative analyses carried out in some sensitive areas, we can state that State Medical Assistance brings incontestable advantages for people’s health [36]. However, its under-utilization, combined with barriers to exercising rights, once they are established, limit the measure’s effectiveness in terms of public health.

## Discussion

Despite the wide range of points of view, current consensus recognizes that, for health and humanitarian reasons, and in line with various ratified conventions,^17^ basic public intervention, at the least, is necessary to give undocumented migrants access to health care. The question is to determine what form this intervention should take. We can identify two stands: one recommends reintroducing undocumented migrants into the common law system^18^ (and thus merging State Medical Assistance with PUMA and CMUc); the other wants to maintain a specific system.

The arguments of the first group are based on considerations linked to the legal framework, public health, and benefits management. In terms of the legal framework, when it comes to basic rights, the Preamble of the French Constitution of 1946 guarantees a principle of equality that cannot exclude a category of the population effectively already residing in France. In this perspective, some lawyers specializing in social rights [37] have observed that the replacement of the residency criterion with that of regular stay in 1993 is in contradiction with the principles of the Constitution^19^ and the initial foundations of the French Social Security system.

In terms of public health, the suppression of State Medical Assistance and the integration of undocumented migrants into common law social protection would result in fewer breaches of rights and therefore fewer breaches of care. In addition, fewer private-sector doctors would refuse to give treatment because they do so less frequently for CMUc beneficiaries than for State Medical Assistance beneficiaries. Lastly, it would simplify procedures for patients and healthcare professionals and avoid lack of knowledge of the measure. Thus, more people would be covered, and better covered.

In terms of benefits management, given that most undocumented people are rejected asylum seekers^20^ who benefited from CMU while their application was being processed, the fact of switching them to another measure incurs administrative costs. Maintaining undocumented people in a common law system that they were previously part of would improve healthcare paths and reduce the consumption of hospital treatment, which is more costly. Lastly, more people would be covered and “emergency care” costs would decrease.

For those who advocate maintaining a specific measure for undocumented migrants, the proposal to bring all people living in economically difficult circumstances under the common law system raises the issue of fairness towards those who come under the national insurance system. If the two types of claimant were subject to the same rights, it would no longer be possible to impose the same administrative obligations, for example, the need to present precise evidence of income. This “unprecedented universality” would not correspond to the principles on which the Social Security system is built [27]. However, analyses of the benefits of maintaining State Medical Assistance are also subject to sometimes contrasting points of view. On the one side, some consider that the current scope of intervention should be maintained at the risk of creating higher costs when situations worsen; on the other side, some advocate restricting the benefit to emergency and priority treatment combined with an obligatory minimum contribution. This latter point is often put forward as an element of fairness towards the French population, most of whom contribute financially to the health system.

Having set out these two positions, it seems to us that reintroducing undocumented migrants into the common law system is more pertinent, both in terms of public health and finances. However, to be conclusive, such a development would require advances in administrative organization and healthcare.

As we have seen, the administrative procedures to establish entitlement are a significant barrier. The supporting documentation required currently varies depending on the region. The question of domiciliation is particularly important.^21^ In addition, people who do not have a fixed home sometimes encounter difficulties in finding structures (usually municipal social services or associations) that accept, or have the capacity, to supply them with an address of domiciliation.^22^ An important step is therefore to study the reasons for this variety of practices and then standardize them with common law practices.^23^

Another step would involve perfecting the path to healthcare by facilitating prevention through consolidating a network of local health centres in areas particularly concerned by the reception of undocumented migrants. These initiatives could also be accompanied by more interpreting services to improve relations between the interested parties and care services. Limited language proficiency appears to be a major obstacle to using the services available.

Cultural mediation is barely utilised in France as it may be perceived by many as contrary to the “republican” model. But it can be useful to promote migrants’ health [38].

It would also seem that the discriminatory practices of some professionals (when establishing the eligibility of potential beneficiaries, domiciliation refusal, treatment refusal, treatment orientation) strongly hinder primary and secondary access to care [15, 39]. Research based on field studies is required to understand the processes that generate these barriers and their impacts on the health of undocumented migrants. Lastly, the fear of expulsion plays a significant role in non-take-up of care. Once again, complementary research would be useful to study representations vis-à-vis migrant populations and the types of relationships that they maintain with the French health system.

Finally, we suggest data collection on migrants’ health at the national level (linked with European indicators for comparison) in order to monitor and assess the impact of policies. For that, we suggest the creation of working groups with the main actors of public health in France (hospitals, NGOs, maternal and child services, school health services, etc.) in order to define relevant and effective indictors.

Beyond these considerations on the concrete operation of the system, it is clear that the State Medical Assistance measure that we have presented currently straddles policies on health and immigration. Changes in welfare protection for undocumented migrants show a move to reduce this cover, not just for financial reasons, but for political reasons linked to regulating immigration [40], which is to the detriment of public health.

This process is more generally implemented in the other measures relating to social rights for foreign nationals [41]. Frenetic legislation on the theme of immigration since the 1980s [7] has developed a categorization of foreign national. This is not a simple mechanical operation free from meaning and social impact [42]. In fact, changes in policies on immigration have reduced the opportunities for migrants to become part of their host society in satisfactory conditions. The consequences are clearly visible in the low ranking of France’s indicators for migrant integration compared with other countries in the OECD area [10], [http://www.mipex.eu/]. This situation indicates negative representations of migrants and those perceived to be migrants, and a constant climate of suspicion [32].

It is interesting to observe more precisely what this implies in the field of health policy and for the professionals who implement it. In general, the question of migrant health is subject to a “fundamental tension” between “legitimate health” and “illegitimate immigration” [43]. Translated into political objectives, this tension results in competition between two sector-based ways of thinking: one aims to reduce the social rights of a share of people living in France, and the other aims at equal rights to healthcare [41]. The rise in force of these opposing views over recent decades has led to a progressive conception of the welfare system to exclude migrants, or even work against them, particularly in the case of measures applied to undocumented migrants [44].

Thus, access to healthcare for undocumented migrants currently creates a dilemma in contemporary democratic systems, by creating tension between citizens’ rights and human rights [45]. However, we have sometimes observed shifts in perspective. Thus, when it comes to admitting sick migrants,^24^ illness, which was a source of suspicion when migrants were part of the labour force following the Second World War, has become a source of social recognition in opening up the right to a residence permit [46]. The issue is no longer about affirming a right to healthcare, but about using health to affirm one’s rights [47]. However, here once again, the context has evolved and the criteria have become stricter. These shifts are not restricted to France and we have observed particularly severe proposed laws in some European countries, termed “security packages”. Their aim is to oblige doctors to inform the police of any undocumented migrant who comes to seek care in their surgery. These proposals have been subject to strong opposition^25^ [45].

In France, some members of the public health community have taken strong positions in recent years on issues connecting health and immigration. For example, the trade union of public health inspectors has underlined the importance of maintaining the independence of public health in the face of immigration policies; and the union of maternal and child welfare doctors has pointed out that “medical ethics are universal”. Some private-sector doctors have given very concrete support to health cover by offering free treatment. Many NGOs, associations and local communities are also active on these key points.

Comparative research at the European level would be useful to better understand the relationship between health and immigration in other countries, to appreciate specific national contexts, the values put forward and how they are expressed in policies, and to understand emerging issues and controversies and how they are dealt with.

## Conclusion

In France, State Medical Assistance has been subject to strong criticism for a number of years. The political aspect of the debate on migration, along with budget constraints, is currently adding to the friction, which combines questions on how to manage immigration with appropriate use of national social efforts. The result is a shifting legal situation that constantly feeds into controversies with changing political directions.

Although State Medical Assistance represents a relatively high cost for the community, it should also be perceived as an expenditure that is likely to avoid other, potentially much higher, costs. To make access to primary care impossible for people often living in very insecure social situations would worsen their health status and could rapidly result in a need for emergency care. Given that society is ethically bound to react in this kind of circumstance, which can threaten people’s lives, it would lead to higher costs than those incurred when diseases are treated at an early stage.

However, State Medical Assistance does not represent a totally satisfactory solution. The stopping of benefits when administrative situations change, the mediocre quality of healthcare paths, and the high levels of non-take-up are all valid reasons for replacing the measure with common law welfare. As we have seen, many stakeholders from diverse backgrounds are aware of the difficulties of the current system and the resulting excess costs and are now speaking out in favour of this solution that we also find relevant. This merger with the common law welfare, less discriminatory, in line with fundamental social rights, would enable a greater continuity and stability in access to rights, a simplification of the management of the scheme, and finally, to do better and cheaper. For a better integration in the national public health policy, we recommend the strengthening of health centres, language proficiency and cultural mediation provision, and the development of protocols on socio epidemiologic data collection.

As the new universal health protection comes into place to unify and rationalize the health cover system, this kind of reform of the State Medical Assistance would be a positive step to reduce stigmatization, reinforce public health and strengthen social cohesion.

## Abbreviations

CMU: Universal Health Cover
CMUc: Complementary Universal Health Cover
OECD: Organization for Economic Co-operation and Development
NGO: Non Governmental Organization
PICUM: Platform for International Cooperation on Undocumented Migrants
IGAS: General Inspectorate of Social Affairs
IGF: General Inspectorate of Finances
GISTI: Group of Information and Support for Migrants
COMEDE: Medical Committee for Exiled Persons
TERRA: Studies and researches on Refugees and Asylum
PUMA: Universal Health Protection
INSEE: National Institute Of Statistics and Economic Studies
INED: French Institute for Demographic Studies
DREES: Direction of Research Studies, Evaluation and Statistics
CNRS: National Centre for Scientific Research

## Footnotes

1 According to the National Institute of Statistics and Economic Studies (INSEE) of France, an “immigré” is someone who is born foreign national in a foreign country. This article uses this definition. We have translated the term “immigré” in “migrant”, because the term “immigrant” has a negative political connotation in English.

2 Optional health insurance pays back the share of expenditure not covered by the Social Security system.

3 30 % of migrant households live in relative poverty, compared to 13 % of other households. This is one of the largest gaps in the OECD area.

4 In 2014, the poverty threshold was 993 euro per month for a single person.

5 The other social risks covered by social protection are: old age, employment, family, housing and exclusion-poverty.

6 Along with health insurance and the state, the other two funders are: optional complementary health insurance (13.5 %) and households (8.5 %).

7 Until 1^st^ January 2016, another, so-called “basic”, CMU existed covering all people of French or foreign nationality residing in France for more than three months in a regular, stable manner and not otherwise covered by a social security scheme. It was free and state funded and benefitted those covered by social security whose annual income was below a specific level (9,601 euro on 31 December 2015). Above this threshold, people had to pay in 8 % of their tax income. This basic CMU was scrapped on 1^st^ January 2016. It is now included in the new universal health - protection universelle maladie (PUMA), which concerns the whole population (excluding undocumented migrants).

8 Prior to this generalization, the condition of regular residency was introduced in 1975 to cover the cost of abortion (condition then removed in 2000) and in 1986 for the payment of family benefits.

9 State Medical Assistance does not cover the cost of therapeutic accommodation, homecare services, or therapeutic trials. Like for CMU, any excess fees are not reimbursed.

10 The “emergency care” measure does not correspond to an individual right to benefits; it takes the form of a fund, financed by the state, through which healthcare establishments can cover the costs of this ethical obligation.

11 Among the best-known reasons are: flight from a country at war, risk of political repression, and situations of extreme poverty.

12 IFOP survey, September 2012: “L’état de l’opinion quatre mois après l’élection présidentielle”.

13 We studied researches mainly from the National Center for Scientific Research (CNRS), the French Institute for Demographic Studies (INED), and the Direction de la recherche, des Etudes, de l’Evaluation et des Statistiques (DREES) form the Ministry of welfare and health

14 For the mast data know, in 2008, a State Medical Assistance beneficiary consumed €1,741 of care, whereas a CMU beneficiary consumed €2,606 [35]

15 Note that this relates to data produced in 2009. The evolution of immigration policies points to a rise in the number of undocumented migrants.

16 A survey carried out in 2007 by Médecins du Monde showed that the refusal rate for treatment was 37 % for State Medical Assistance beneficiaries and 10 % for CMU beneficiaries.

17 European Convention of Human Rights and Fundamental Freedoms of 4 October 1950, European Social Charter on the Right to Social and Medical Assistance (Article 13 of the International Pact on Civil and Political Rights – law of 15 June 1980), Convention on the Rights of the Child of 20 November 1989.

18 This development has been recommended in recent years by humanitarian associations, the National Council for policies to combat poverty and social exclusion, the Rights Commissioner and the IGAS in its 2010 report.

19 Based on this reflection, as a reminder, in 1993, the Constitutional Council only accepted that the new law was in conformity with the provisions of the 1946 Preamble because of the power granted to the minister in charge of social assistance to allow, by way of derogation, foreign nationals to benefit from medical assistance beyond the conditions for documented residency.

20 Since 1987, the rate of obtaining refugee status in France has varied from 15 % to 30 %. For information, in 2015, 79,126 asylum requests were filed and 19,447 were accepted. (Data from the Home Office and the French Office for the Protection of Refugees and Stateless People).

21 For State Medical Assistance, domiciliation issues appear to represent 30 % of non-access to rights [16]

22 Municipalities, via their social services, are obliged to provide an address of domiciliation to anyone without a stable address who has a connection to the town.

23 People in totally regular situations but with no fixed address are confronted with the same domiciliation difficulties to claim their rights.

24 This measure (residence permit for “humanitarian reasons”), in force since 1994, is aimed at people whose state of health requires treatment that they cannot obtain in their country of origin. The criteria for granting it have become stricter in recent years.

25 Opponents of the draft law took up the slogan: “We are doctors and nurses, not spies”.
